# Effect of Levodopa on Reward and Impulsivity in a Rat Model of Parkinson’s Disease

**DOI:** 10.3389/fnbeh.2017.00145

**Published:** 2017-08-08

**Authors:** Miguel M. Carvalho, Filipa L. Campos, Mariana Marques, Carina Soares-Cunha, Nikolaos Kokras, Christina Dalla, Hugo Leite-Almeida, Nuno Sousa, António J. Salgado

**Affiliations:** ^1^Life and Health Sciences Research Institute (ICVS), School of Health Sciences, University of Minho, Campus de Gualtar Braga, Portugal; ^2^ICVS/3B’s, PT Government Associate Laboratory Guimarães, Portugal; ^3^Department of Pharmacology, Medical School, National and Kapodistrian University of Athens Athens, Greece; ^4^First Department of Psychiatry, Medical School, National and Kapodistrian University of Athens Athens, Greece

**Keywords:** Parkinson’s disease, dopamine dysregulation syndrome, impulse control disorders, 6-OHDA, levodopa

## Abstract

The use of dopamine replacement therapies (DRT) in the treatment of Parkinson’s disease (PD) can lead to the development of dopamine dysregulation syndrome (DDS) and impulse control disorders (ICD), behavioral disturbances characterized by compulsive DRT self-medication and development of impulsive behaviors. However, the mechanisms behind these disturbances are poorly understood. In animal models of PD, the assessment of the rewarding properties of levodopa (LD), one of the most common drugs used in PD, has produced conflicting results, and its ability to promote increased impulsivity is still understudied. Moreover, it is unclear whether acute and chronic LD therapy differently affects reward and impulsivity. In this study we aimed at assessing, in an animal model of PD with bilateral mesostriatal and mesocorticolimbic degeneration, the behavioral effects of LD therapy regarding reward and impulsivity. Animals with either sham or 6-hydroxydopamine (6-OHDA)-induced bilateral lesions in the *substantia nigra pars compacta* (SNc) and ventral tegmental area (VTA) were exposed to acute and chronic LD treatment. We used the conditioned place preference (CPP) paradigm to evaluate the rewarding effects of LD, whereas impulsive behavior was measured with the variable delay-to-signal (VDS) task. Correlation analyses between behavioral measurements of reward or impulsivity and lesion extent in SNc/VTA were performed to pinpoint possible anatomical links of LD-induced behavioral changes. We show that LD, particularly when administered chronically, caused the development of impulsive-like behaviors in 6-OHDA-lesioned animals in the VDS. However, neither acute or chronic LD administration had rewarding effects in 6-OHDA-lesioned animals in the CPP. Our results show that in a bilateral rat model of PD, LD leads to the development of impulsive behaviors, strengthening the association between DRT and DDS/ICD in PD.

## Introduction

Parkinson’s disease (PD) is a chronic neurodegenerative disorder affecting the dopaminergic system, causing significant neuronal loss in the *substantia nigra pars compacta* (SNc, or A9), and in the ventral tegmental area (VTA, or A10; Hirsch et al., 1988; German et al., 1989; Parkinson, 2002), and characterized by a number of different motor and non-motor symptoms (Jankovic, 2008; Obeso et al., 2008; Chaudhuri and Schapira, 2009; Dirnberger and Jahanshahi, 2013). Dopamine replacement therapies (DRT), namely levodopa (LD) and dopamine agonists (DAg), can significantly ameliorate motor and non-motor symptoms of PD (Maricle et al., 1995; Antonini et al., 2009; Hauser, 2009; Seppi et al., 2011). However, in some patients, chronic DRT leads to the emergence of addictive and/or impulsive behaviors. Abuse of DRT, particularly LD, a condition known as dopamine dysregulation syndrome (DDS), is characterized, among other symptoms, by the development of compulsive self-medication behaviors (Giovannoni et al., 2000; Pezzella et al., 2005). In turn, impulse control disorders (ICD), mainly associated with DAg, are characterized by the development of several “behavioral addictions” (Potenza et al., 2007), namely pathological gambling, hypersexuality and eating and buying/shopping compulsions (Molina et al., 2000; Klos et al., 2005; Pezzella et al., 2005; Weintraub et al., 2010). The particular association between each set of behavioral disturbances and a class of DRT could suggest independent mechanisms underlying DDS and ICD, however this is not fully understood, as there is evidence supporting a role for LD in increasing the risk of ICD when combined to DAg, compared to DAg monotherapy (Weintraub et al., 2010). Regardless of the fact that the causal mechanisms for DDS and ICD are unknown, it is interesting to observe that they share many behavioral features, including a compulsive pattern of DRT abuse (for DDS) and engagement in “behavioral addictions” (for ICD; Giovannoni et al., 2000; Voon et al., 2017), the hedonic value that DRT abuse and “behavioral addictions” can hold (Giovannoni et al., 2000; Evans et al., 2009); and the development of withdrawal symptoms if LD or DAg dosages are reduced, or if engagement in “behavioral addiction” is restricted (Giovannoni et al., 2000; Evans et al., 2009; Pondal et al., 2013). Remarkably, both of these syndromes also share behavioral and functional similarities with substance addictions or impulsive behaviors in healthy individuals (Voon and Fox, 2007; Dagher and Robbins, 2009). Based on this, and in accordance with a theory of dopamine (DA) “overdose”, DDS and ICD in PD have been linked to an excessive dopaminergic stimulation of the relatively spared mesocorticolimbic pathway caused by DRT (Dagher and Robbins, 2009; O’Sullivan et al., 2009; Voon et al., 2017). Indeed, in the course of PD, the mesocorticolimbic pathway, originating in the VTA (area A10) is less affected than the mesostriatal pathway (German et al., 1989), and several reports have shown a causal link between the specific activation of this pathway and reward-seeking behaviors (Tsai et al., 2009; Pascoli et al., 2015). Such overstimulation is believed to cause oversensitization of brain reward circuits, leading to a heighten reward salience in PD patients, together with insensitivity to negative outcomes (Dagher and Robbins, 2009; O’Sullivan et al., 2009; Voon et al., 2017). Simultaneously, a disruption of cognitive inhibitory networks can impair impulse control (O’Sullivan et al., 2009; Voon et al., 2017). Support for this hypothesis is found in human studies showing that in PD patients, DRT can significantly impair reward-based learning, making patients overvalue positive rewards while desensitizing them to risk or negative outcomes (Bódi et al., 2009; Housden et al., 2010; Voon et al., 2010a, 2011), and foster impulsive responding (Housden et al., 2010; Voon et al., 2010b).

Few studies have tried replicating features of DDS/ICD in animal models of PD, and indeed they are essential for understanding the impact of DRT upon parkinsonian dopaminergic networks and how they may lead to the development of DDS/ICD (Cenci et al., 2015). However, some studies have shown the rewarding effects of specific DAg in rodent models of PD, measured with the conditioned place preference (CPP) paradigm (Riddle et al., 2012; Ouachikh et al., 2013, 2014; Zengin-Toktas et al., 2013). DAg, specifically pramipexole, was also found to cause, in models of PD, impulsive behaviors in a probability-discounting task (Rokosik and Napier, 2012), but also waiting and motor impulsivity (Engeln et al., 2016). In the case of LD, it was also shown that it can have rewarding properties in SNc-lesioned rats (Engeln et al., 2013b), though this finding was inconsistent with another study, which found no effect in animals with bilateral mesostriatal and mesocorticolimbic degeneration (Zengin-Toktas et al., 2013). Evidence of the effects of LD upon measures of impulsive behavior has been more limited, although a recent study described no effects of LD in both motor and waiting impulsivity in animals with SNc dopaminergic lesions (Engeln et al., 2016). Additionally, it is still poorly understood how the chronic effects of LD, in contrast to its acute effects, may differently affect PD dopaminergic pathways and lead to the development of DDS/ICD-like behaviors.

To further address the rewarding effects of different regimes of LD treatment but also their impact upon measures of impulsive behavior, we have used an animal model of PD with bilateral 6-hydroxydopamine (6-OHDA) SNc/VTA (A9/A10) lesions, mimicking the degenerative profile of human PD. We evaluated the potential of both acute and chronic administration of LD to cause the emergence of impulsive behaviors, expressed as premature responding in a variable delay-to-signal task (VDS; Leite-Almeida et al., 2013). Moreover, we employed the CPP paradigm to assess the rewarding nature of acute or chronic LD treatments in our model. Finally, we performed individual anatomical analyses of lesion extent to identify correlates of these behaviors, which may be important for the comprehension of the pathophysiology of DDS/ICD in human PD.

## Materials and Methods

### Animals and 6-OHDA Lesions

Wistar-Han male rats (Charles River, Barcelona), 11 weeks old were housed, two per cage, under standard laboratory conditions: 12 h light–dark cycle, 22°C room temperature, 55% relative humidity, food and water available *ad libitum*. When required, food availability was restricted to 1 h/day (19.00–20.00), with weight regularly monitored to prevent losses 15% below of initial weight. Behavioral assessment was performed between 09.00 AM and 16.00 PM, during the light part of the cycle. Previous consent was obtained from the Portuguese national authority for animal experimentation, Direção-Geral de Alimentação e Veterinária. Animals were kept and handled in accordance with the guidelines for the care and handling of laboratory animals in the Directive 2010/63/EU of the European Parliament and of the Council.

6-OHDA lesions were performed under ketamine-medetomidine anesthesia (75 mg/Kg: 0.5 mg/Kg, i.p.). Thirty minutes before 6-OHDA injections the animals were treated with desipramine hydrochloride (Sigma, San Antonio, TX, USA), 15 mg/Kg, i.p., to avoid uptake of 6-OHDA by noradrenergic neurons. Bilateral sham (sham group, *n* = 31) or 6-OHDA hydrochloride (Sigma; 6-OHDA group, *n* = 33) lesions were made in both SNc and VTA. SNc and VTA coordinates related to Bregma and according to Paxinos and Watson (1998): SNc: AP = −5.2 mm; ML= ± 2.2 mm; DV = −7.7 mm. VTA: AP = −5.2 mm; ML= ± 0.8 mm; DV = −8.2 mm. The incisor bar was set at −3.3 mm. Sham animals received 2 μl of 0.2 μg/μl ascorbic acid in 0.9% NaCl. 6-OHDA animals received 2 μl of 1.0 μg/μl 6-OHDA hydrochloride with 0.2 μg/μl ascorbic acid in 0.9% NaCl. All injections were performed with a 0.5 μl/min flow, and the needle was left in place for 4 min after each of the four injections.

### Experimental Design

**Figure 1:**
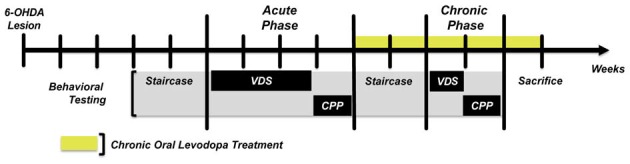
Graphic representation of the experimental design. Staircase—Forelimb skilled motor function. VDS, Variable delay-to-signal—Impulsive responding. All sham and 6-OHDA animals performed the task in both behavioral assessment phases. CPP, Conditioned Place Preference—Positive reinforcement. For CPP, sham and 6-OHDA groups were divided in two subgroups, one performing the task in the acute phase and the other on the chronic phase. This was done to avoid possible conditioning memories created during the acute phase from biasing conditioned responses in the chronic phase.

The experimental protocol (Figure 1) was initiated 3 weeks post-surgery. After an initial assessment of motor function with the staircase test, the protocol continued with two blocks of behavioral assessment. In the first block (acute phase) we assessed the effects of an acute LD treatment in the development of impulsive behavior using the VDS task, and of LD reward using the CPP. We then initiated chronic LD treatment to all the animals enrolled in the study (sham and 6-OHDA), and after 1 week of treatment motor function was reassessed. After 2 weeks of chronic LD treatment we performed the second block of behavioral assessment (chronic phase), using the same tasks as in the first block. Chronic LD treatment was continued throughout the second block of behavioral assessment and until the end of the study.

### LD Treatment

Sham and 6-OHDA animals were exposed to both acute and chronic LD according to the experimental design (Figure 1). For acute LD treatment, Sinemet^®^ tablets (Sinemet^®^, 100/25 levodopa/carbidopa, Merck, Sharp and Dohme, S.p.A, Italy) were crushed in water and given by oral gavage, 12 mg/Kg, 1 h before behavioral assessment. Oral gavage was chosen as route of administration to more closely replicate the human therapeutic setting. Treatment doses were based on the therapeutic range of LD reported for a unilateral lesion model of PD (Lindner et al., 1996), but adjusted to avoid severe dyskinesias in our model with bilateral dopaminergic degeneration. During chronic LD treatment both sham and 6-OHDA animals received oral LD, 12 mg/Kg, twice daily, one dose between 08.00 AM and 12.00 AM and another between 16.00 PM and 20.00 PM, until the end of the study. In the second block of behavioral assessment the animals also performed the tests “ON” LD, receiving the morning treatment 1 h before behavioral assessment.

### Behavioral Assessment

#### Staircase Test

To assess skilled forelimb motor function, we performed the staircase test (Model 80300, Campden Instruments, UK; Klein and Dunnett, 2012). Before 6-OHDA lesions, food-deprived animals were presented with the food pellets in their home cage and trained in one daily session for five consecutive days. On day 1 the animals were habituated for 5 min to the box. On day 2 the animals were placed 10 min in the box and presented with pellets on each side of the stair and along the central plinth. From days 3 to 5 the animals were placed in the box for 15 min with food pellets present only on the staircases. Staircase testing was performed in food-deprived animals during seven consecutive days, with a daily 15 min session. During each session forelimb function was measured as success rate, i.e., percentage of total pellets successfully retrieved, and results were plotted for each group and each testing day. Testing sessions were conducted after lesion, to assess the effect of 6-OHDA injections in motor function, and a randomly selected sample of sham and 6-OHDA animals was retested after 1 week of chronic LD treatment (Figure 1), to determine the therapeutic efficacy of the LD doses used in the study. This retest was performed “ON” LD, 1 h after treatment administration. Importantly, after LD administration some 6-OHDA animals (*n* = 5) developed noticeable forelimb dyskinesias in the form of involuntary motor jerks and were incapable of accomplishing the staircase task. To avoid this bias on the general efficacy of LD to improve motor function, both pre and post-treatment data of these animals was removed.

#### Variable Delay-to-Signal

To assess impulsive behavior we performed the VDS task (Leite-Almeida et al., 2013). Animals were kept food-deprived during the whole protocol, which included habituation, training and testing phases. The protocol was performed in a nearly square shaped (25 × 25 cm) 5-hole operant chamber (OC; TSE Systems GmbH, Germany). Five square apertures (2.5 × 2.5 cm; #1–#5) equipped with a 3W light bulb and infrared photobeams to detect movements were distributed in a slightly curved wall. On the opposite wall there was another aperture (#6), also equipped with light and photobeams, connected to a food dispenser. Above aperture #6 a houselight illuminated the OC. OCs were placed inside a sound-attenuating chamber, with electrical fans providing ventilation and white noise.

Habituation phase took place in two consecutive days, each with two daily sessions with a 5 h interval. On the first two sessions the animals were placed inside the boxes for 15 min, in darkness, and presented with food pellets (45 mg; BioServ Inc., Flemington, NJ, USA) in aperture #6; apertures #1–#5 were blocked with a metallic cap. On sessions 3 and 4, the animals were placed for 30 min inside the box, with lights #3, #6 and house-light turned on. Food pellets were freely available on #3 and #6 (apertures #1, #2, #4 and #5 were blocked in this and in the upcoming training and testing sessions). The training sessions started the next day and were performed once a day. Each training session started with the delivery of a food pellet. Pellet collection in #6 triggered a 3 s intertrial interval (ITI)—delay period—that was followed by a response period in which #3 light was on. Nosepokes in aperture #3 within 60 s were rewarded with a food pellet and a new ITI occurred. Responses in the delay period—premature responses—were punished with the houselight turning off (5 s) and absence of reward. Training sessions were carried until 100 trials were completed or until 30 min had elapsed, on several consecutive days until average premature responding stabilized. Training sessions lasted 11 days in the acute phase and four in the chronic phase. Animals failing to complete 100 trials in the 30 min limit at the end of the training period were removed from the task. At the end of the training period animals were exposed to the single VDS testing day, during which the animals were subjected to 25 initial trials with a 3 s delay period, followed by 70 trials of randomly selected 6 s or 12 s delay periods. In the testing day, premature responses, executed before the delay period had elapsed and #3 light was turned on, were not punished and were recorded as a measure of impulsivity. Both sham and 6-OHDA animals were exposed to the complete task, on both behavioral assessment phases (Figure 1). In the acute phase, training was performed without any treatment and on the testing day the animals in each group were randomly treated with either LD or vehicle, 1 h before starting the test. In the chronic phase, training sessions were performed “OFF” LD, with the animals receiving their daily LD treatment only after ending that day’s training session. In the testing day the animals received the same treatment as in the acute phase, 1 h before starting the test.

#### Conditioned Place Preference

After the end of the VDS task animals were returned to an *ad libitum* food regime. The rewarding nature of LD was assessed with the CPP paradigm during eight consecutive days. The apparatus consisted of a central gray box, connected on each side by a lateral door to two boxes, distinct in both color (black/white) and floor texture (grid/wire mesh; MED-CPP-013, Med Associates, Inc., St. Albans City, VT, USA). On day 1 the animals were placed for 5 min in the gray box for habituation, after which the lateral doors were opened, allowing the animals to freely explore both the white and the black box for 15 min. The box where the animal spent more time was considered the preferred one. On days 2–7 the animals were conditioned, based on their initial preference. On days 2, 4 and 6 the animals received water, orally, 1 h before being placed in the preferred box for 20 min. On days 3, 5 and 7 the animals received LD, orally, 1 h before being placed in the non-preferred box for 20 min. On day 8, after 5 min habituation in the gray box the animals were allowed to freely explore all the boxes. Reward-seeking behavior was assessed by comparing time spent in the assigned LD box during pre-conditioning and post-conditioning stages of the task. Due to the persistence of possible conditioned memories between the two behavioral assessment phases we divided our experimental groups in two. One group of both sham and 6-OHDA animals were exposed to the CPP in the acute phase and the other group was used in the chronic phase (Figure 1).

### TH Immunohistochemistry and TH^+^ Cell Counts

At the end of the experimental protocol animals were deeply anesthetized with sodium pentobarbital and transcardially perfused with ice-cold 0.9% NaCl. The animals were decapitated and the heads were immersed in liquid nitrogen for 10 s. The brains were removed and cut into two blocks. The anterior block was used for macrodissection of the dorsal striatum (dSTR) and nucleus accumbens (NAcc). The samples were stored at −80°C until being processed for high performance liquid chromatography (HPLC) measurements of DA concentrations. The posterior block, containing the ventral mesencephalon, was post fixed in 4% PFA for 3 days (room temperature) and then stored in 8% sucrose (4°C) until further use. Five series of coronal sections of the mesencephalon, 30 μm thick, were obtained using a vibratome (VT1000S, Leica, Germany), and one series was used for tyrosine hydroxylase (TH) immunohistochemistry. Briefly, inhibition of endogenous peroxidase was performed by immersing the slices in 1× PBS with 3% H_2_O_2_ for 20 min. Slices were then blocked with 5% fetal calf serum in 1× PBS for 2 h and incubated over-night at 4°C with rabbit anti-rat TH primary antibody (Millipore, Billerica, MA, USA, 1:2000 in 2% fetal calf serum in 1× PBS-T). The slices were then incubated 30 min with a biotinylated secondary anti-rabbit antibody (LabVision, Fremont, CA, USA) then 30 min with an Avidine/Biotine complex (LabVision). Antigen visualization was performed with 3,3í-diaminobenzidine tetrahydrochloride (DAB, Sigma; 25 mg DAB in 50 ml Tris–HCl 0.05 M, pH 7.6 with 12.5 μl H_2_O_2_).

**Figure 2:**
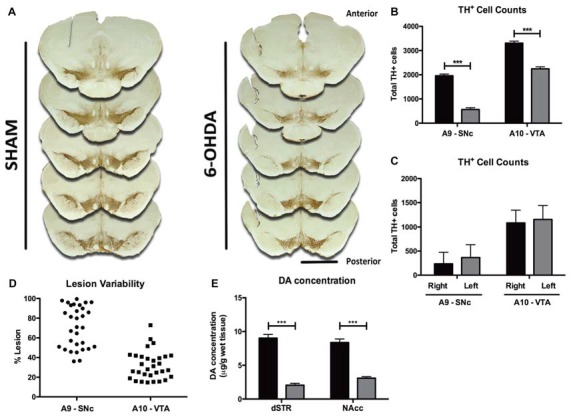
Histological and neurochemical characterization of lesion extent. **(A)** Representative photomicrographs of the five tyrosine hydroxylase (TH)-labeled slices selected along the anterior-posterior axis for histological characterization of lesion extent. Scale bar = 2 mm. **(B)** Quantification of the total number of TH^+^ cells counted bilaterally in A9 and A10 on the five selected slices of both sham (black bars, *n* = 10) and 6-OHDA animals (gray bars, *n* = 31). **(C)** Quantification of the number of TH^+^ cells in each hemisphere of both A9 and A10 in 6-OHDA animals (*n* = 31). **(D)** Representation of 6-OHDA lesion variability in A9 and A10 regions (*n* = 31). **(E)** Measurement of DA content in the dorsal straitum (dSTR, sham: black bars, *n* = 31; 6-OHDA: gray bars, *n* = 29) and nucleus accumbens (NAcc sham: black bars, *n* = 31; 6-OHDA: gray bars, *n* = 31). Data presented as mean ± SEM. ***p < 0.0001.

Quantification of lesion extent was made by TH^+^ cell counts. Five identical TH-labeled slices, along the anterior-posterior axis of the mesencephalon and containing both the A9 and A10, were selected per animal based on anatomical references (Figure 2). With a bright-field microscope (BX51, Olympus, USA), equipped with a digital camera (PixeLINK PL-A622, CANIMPEX Enterprises Ltd., Halifax, NS, Canada), we used Visiomorph™ (V2.12.3.0, Visiopharm, Denmark) software to draw the boundaries of areas A9 and A10 (Paxinos and Watson, 1998). Area A9 comprised all regions of SNc, while A10 area included the VTA, parabrachial nucleus, paranigral nucleus, rostral and caudal linear nuclei and interfascicular nucleus, as previously described (Rodríguez et al., 2001; Bentivoglio and Morelli, 2005). All A9 and A10 TH^+^ cells were counted in both hemispheres for each of the five slices of sham and 6-OHDA animals and summed for each area, per animal. Lesion extent in A9 and A10 for each 6-OHDA animal is expressed as % of total TH^+^ cells lost in each area, compared to the average total of TH^+^ cells in sham animals.

### High Performance Liquid Chromatography

DA tissue levels in dSTR and NAcc were measured using reverse phase ion pairing high performance liquid chromatography with electrochemical detection (HPLC-ED) as previously described (Kyratsas et al., 2013; Novais et al., 2013) with minor modifications. Specifically, samples were weighed, homogenized and deproteinized in 0.1 N perchloric acid solution (Applichem, Darmstadt, Germany) containing 7.9 mM Na_2_S_2_O_5_ and 1.3 mM Na_2_EDTA (Riedel-de Haën AG, Seelze, Germany), then centrifuged at 4°C for 45 min at 20,000 g and the supernatant was stored at −80°C until analysis. The mobile phase consisted of a 50 mM phosphate buffer at pH 3.0, with 300 mg/L 5-octylsulfate sodium salt as the ion pairing reagent, 20 mg/L Na_2_EDTA (Riedel-de Haën AG) and 8%–12% v/v acetonitrile (Merck, Darmstadt, Germany) The working electrode of the electrochemical detector was set at +800 mV. The column used was an Aquasil C18 HPLC Column, 250 mm × 4.6 mm, 5 μm Particle Size (Thermo Electron, UK). As previously (Bessinis et al., 2013; Melo et al., 2015), the quantification of DA was done by comparing the area under the curve against known external reference standards using appropriate HPLC software (Clarity v.7 DataApex, Prague, Czech Republic). The detection limit was 1 pg/20 ul of sample volume and results are expressed as microgram of DA per gram of wet tissue.

### Statistical Analysis

All data presented as mean ± SEM and analyzed using GraphPad Prism^®^ 5.0b (GraphPad Software Inc., San Diego, CA, USA) and IBM^®^ SPSS^®^ Statistics 22 (IBM Corporation, Armonk, NY, USA). Histological neurochemical and CPP data were analyzed with Student’s *T* test. The results of the staircase and VDS tests were performed using mixed-design ANOVA and Kruskal-Wallis test, followed by Dunn’s *post hoc* test. Analyses of correlation between degree of lesion and measures of impulsive responding and degree of CPP conditioning were performed with the Pearson’s test. Statistical significance set at *p* < 0.05.

## Results

### Histological and Neurochemical Lesion Characterization

Histological analysis of brain slices showed that 6-OHDA animals displayed a marked anterior-posterior reduction in TH staining in A9 and A10 on both hemispheres (Figure 2A). Quantitatively, 6-OHDA injections caused a significant loss of ~70% of TH^+^ cells in area A9 (*t* = 10.24, *p* < 0.0001), and of ~32% in area A10 (*t* = 6.698, *p* < 0.0001; Figure 2B), with no differences in lesion extent between hemispheres in either brain area (Figure 2C; *t* = 1.452, *p* = 0.1517 for A9, *t* = 1.046, *p* = 0.2998 for A10). Importantly, 6-OHDA lesions showed some degree of variability, ranging from 36% to 99% in the SNc and from 14% to 73% in the VTA (Figure 2D). To further characterize the dopaminergic degeneration induced by 6-OHDA lesions, we performed HPLC measurements of DA content in the major projection targets of A9 and A10 DA neurons—dSTR and nucleus NAcc (Figure 2E). Consistent with the dopaminergic lesions induced in A9 and A10, 6-OHDA animals presented significant decreases of DA content in both areas, compared to sham animals (dSTR: *t* = 11.030, *p* < 0.0001; NAcc: *t* = 9.252, *p* < 0.0001).

### Assessment of Motor Function and Effect of LD Therapy

**Figure 3:**
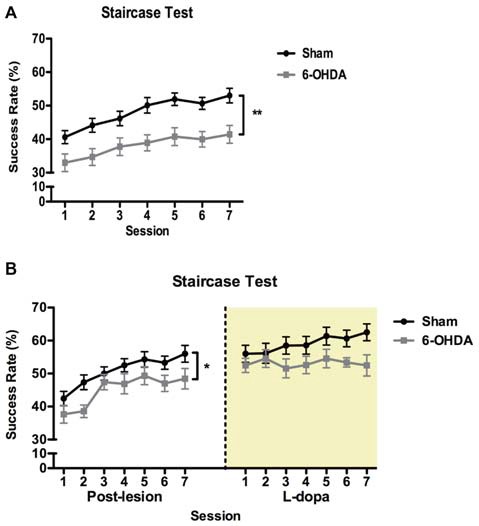
Effect of 6-OHDA lesions and levodopa (LD) treatment on skilled forelimb motor function. **(A)** Longitudinal post-lesion assessment of staircase. 6-OHDA, *n* = 30; sham, *n* = 28. **(B)** Reassessment of motor performance during LD treatment in a subset of 6-OHDA (*n* = 11) and sham (*n* = 20) animals tested in **(A)**. Data presented as mean ± SEM. *p < 0.05; **p < 0.01.

In the staircase test (Figure 3) repeated-measures analysis showed that performance was significantly affected by session (*F*_(1,57)_ = 8.616, *p* = 0.005), indicating an overall improvement of success rate with repeated sessions (Figure 3A). Importantly, 6-OHDA-lesioned animals developed severe motor impairments in forelimb function compared to sham animals (*F*_(6,342)_ = 13.897, *p* < 0.001; Figure 3A). There was no effect of the interaction between these two factors (*F*_(6,342)_ = 0.545, *p* = 0.774).

One week after initiating LD treatment we retested a subset of both 6-OHDA and sham animals, 1 h after the morning dose of LD, to evaluate the therapeutic relevance of the LD treatment used in our study (Figure 3B). In this subset of animals, we confirmed that in the post-lesion period motor performance was significantly affected by session (*F*_(6,174)_ = 15.281, *p* < 0.0001), with 6-OHDA-lesioned animals displaying a significantly lower success rate in retrieving food pellets (*F*_(1,29)_ = 4.356, *p* = 0.046), and no interaction between the factors (*F*_(6,174)_ = 0.7205, *p* = 0.634). With treatment, we observed no significant differences in motor performance between sham and 6-OHDA animals (*F*_(1,29)_ = 3.361, *p* = 0.077). Session (*F*_(6,174)_ = 1.110, *p* = 0.359) and the interaction between the factors were also not significant (*F*_(6,174)_ = 1.070, *p* = 0.382).

### Effect of Acute and Chronic LD Treatment on Premature Responses in the VDS

#### Acute Phase

**Figure 4:**
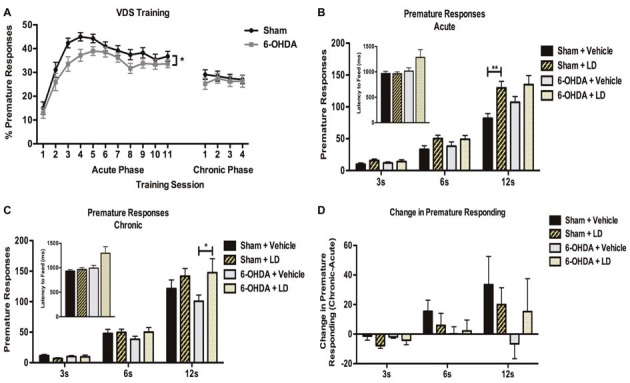
Effect of acute and chronic LD treatments in premature responding in the VDS. **(A)** Behavior performance in the training phase of the VDS protocol for both 6-OHDA (*n* = 28) and sham (*n* = 31). **(B)** Comparison of premature responding between sham and 6-OHDA animals acutely treated with LD (sham + LD, *n* = 15; 6-OHDA + LD, *n* = 13), and their respective vehicle controls (sham + vehicle, *n* = 8; 6-OHDA + vehicle, *n* = 13) for each type of delay trials. Inset indicates average latency to feed for all groups during test. **(C)** Effects of chronic LD treatments on premature responding on sham and 6-OHDA treated with LD (sham + LD, *n* = 15; 6-OHDA + LD, *n* = 14); and their respective vehicle controls (sham + vehicle, *n* = 13; 6-OHDA + vehicle, *n* = 13). Inset indicates average latency to feed for all groups during test. **(D)** Comparison between premature responding in acute and chronic phases of testing (sham + LD, *n* = 15; sham + vehicle, *n* = 8; 6-OHDA + LD, *n* = 13; 6-OHDA + vehicle, *n* = 13). Data presented as mean ± SEM. *p < 0.05; **p < 0.01.

In the training phase of VDS task (Figure 4) both sham and 6-OHDA groups acquired a stable performance (Figure 4A). Repeated-measures analysis showed a significant within-group effect of session day in the number of premature responses (*F*_(10,580)_ = 40.796, *p* < 0.0001). Curiously, the analysis of between-group effects showed that in the overall analysis sham rats displayed a higher % of trials prematurely interrupted than 6-OHDA-lesioned (*F*_(1,58)_ = 3.990, *p* = 0.05). There was no effect of the interaction between the factors (*F*_(10,580)_ = 0.732, *p* = 0.695). On the VDS test session (Figure 4B), our analysis showed a significant effect of delay (*F*_(2,92)_ = 281.631, *p* < 0.0001) and treatment (*F*_(1,46)_ = 9.757, *p* = 0.003) on the number of premature responses as well as an interaction between the factors (*F*_(2,92)_ = 7.855, *p* = 0.001), i.e., LD-treated groups displayed decreased tolerance to delay. Premature responses were not significantly affected by lesion (*F*_(1,46)_ = 0.982, *p* = 0.327), and there were no lesion × delay (*F*_(2,92)_ = 1.766, *p* = 0.177), lesion × treatment (*F*_(1,46)_ = 0.693, *p* = 0.409), or delay × lesion × treatment (*F*_(2,92)_ = 0.500, *p* = 0.608) interactions. Pairwise comparisons between each LD group and their respective vehicle controls showed that only in the 12 s delay the acute LD treatment caused a significant increase in the number of premature responses in the sham group (*p* < 0.05) and a marginal increase in 6-OHDA animals (*p* = 0.08). Additionally, we observed no significant effects of LD treatment on motor behavior, measured with the latency to feed (*H* = 1.603, *p* = 0.659), with *post hoc* pairwise comparisons showing no significant differences between groups (*p* > 0.05; Figure 4B, inset).

#### Chronic Phase

In the training sessions of the VDS during the chronic phase (Figure 4A) both experimental groups presented a similar stable performance, with no significant effects of session day (*F*_(3,174)_ = 0.430, *p* = 0.732), lesion (*F*_(1,58)_ = 0.300, *p* = 0.586), or the interaction of the two factors (*F*_(3,174)_ = 0.666, *p* = 0.574). Data analysis of the VDS (Figure 4C) revealed that delay (*F*_(2,102)_ = 219.283, *p* < 0.0001) and delay × treatment (*F*_(2,102)_ = 5.241, *p* = 0.007) had again a significant effect on premature responding. In contrast, premature responses were not significantly affected by lesion (*F*_(1,51)_ = 0.265, *p* = 0.609), treatment (*F*_(1,51)_ = 2.920, *p* = 0.094), delay × lesion (*F*_(2,102)_ = 0.247, *p* = 0.782), lesion × treatment (*F*_(1,51)_ = 0.833, *p* = 0.366) or delay × lesion × treatment (*F*_(2,102)_ = 0.505, *p* = 0.605). Pairwise comparisons for each of the delays showed that chronic LD intake only caused a significant increase in the number of premature responses in 6-OHDA animals during the 12 s delay (*p* < 0.05). Again, we observed no significant effects of LD treatment on latency to feed (*H* = 6.266, *p* = 0.099), with no significant differences in *post hoc* pairwise comparisons (*p* > 0.05; Figure 4C, inset). To further investigate the chronic effects of LD treatment we next compared the chronic phase to the acute phase performance for each of the groups (Figure 4D). This analysis showed that, for the larger delay, chronic exposure to LD increased, but not significantly (*p* > 0.05), the number of premature responses in all groups, except in 6-OHDA-lesioned animals treated with vehicle.

### Rewarding Effects of Acute and Chronic LD Treatment in the CPP

#### Acute Phase

**Figure 5:**
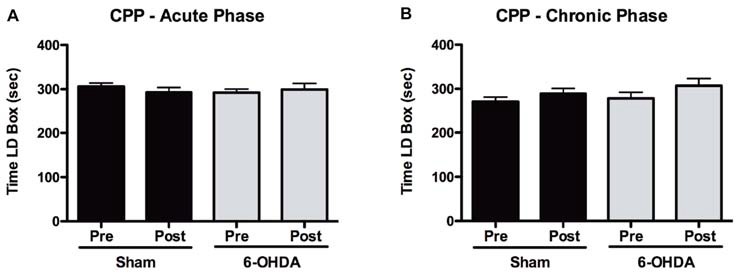
Assessment of positive reinforcement of acute and chronic LD treatments with the CPP.** (A)** Time spent in the LD-paired box during pre and post conditioning stages of the CPP for both sham (*n* = 15) and 6-OHDA-lesioned animals (*n* = 17), after acute LD treatment. **(B)** Time spent in the LD-paired box during pre and post conditioning stages of the CPP for both sham (*n* = 16) and 6-OHDA-lesioned animals (*n* = 16), after exposure to a period of chronic LD. Data presented as mean ± SEM.

Our results from the CPP test (Figure 5) showed that in response to an acute exposure to LD, animals with 6-OHDA lesions had no significant increase in the time spent in the LD-paired compartment (*t* = 0.472, *p* = 0.643; Figure 5A). Similarly, LD treatment had no significant effect in the time spent by sham-lesioned animals in the LD box (*t* = 0.864, *p* = 0.402).

#### Chronic Phase

After a period of 2 weeks of chronic LD treatment a second group of both sham and 6-OHDA-lesioned animals were exposed to the CPP protocol (Figure 5B). Again, we observed that LD did not cause any significant changes in the time that either sham (*t* = 1.239, *p* = 0.234) or 6-OHDA-lesioned (*t* = 1.226, *p* = 0.239) animals spent in the LD-paired box.

### Correlations between Lesion Extent and Behavior Measures

Taking advantage of the observed variability in the individual extent of dopaminergic lesions in 6-OHDA animals, we analyzed potential associations between lesion severity and LD-induced phenotypes in both VDS and CPP tasks. We observed a significant negative association between the number of premature responses of the 6-OHDA + vehicle group in the acute phase and total and right hemisphere A9 lesion extent (*r* = −0.580, *p* = 0.048, for total A9 lesion; *r* = −0.675, *p* = 0.016, for right hemisphere A9 lesion; Table 1). We performed similar analyses between lesion extent and the difference in the time spent in LD box from pre to post-conditioning sessions in the CPP (Table 2) but we found no significant correlations (*p* > 0.05).

**Table 1:** Correlation analysis between A9/A10 lesion extent and premature responses during the 12 s delay trials of the variable delay-to-signal (VDS).

	**Lesion extension**					
	**6-OHDA + Vehicle**					
**Premature responses 12 s**	**A9 lesion**	**A9 lesion right**	**A9 lesion left**	**A10 lesion**	**A10 lesion right**	**A10 lesion left**
Acute phase						
Pearson *r*	**−0.580**	**−0.675**	−0.203	−0.395	−0.293	−0.340
*p*-value	**0.048**	**0.016**	0.528	0.204	0.355	0.280
Chronic phase						
Pearson *r*	−0.108	−0.495	0.311	0.150	−0.173	0.366
*p*-value	0.740	0.102	0.326	0.642	0.5912	0.241
	**6-OHDA + LD**					
**Premature responses 12 s**	**A9 lesion**	**A9 lesion right**	**A9 lesion left**	**A10 lesion**	**A10 lesion right**	**A10 lesion left**
Acute phase						
Pearson *r*	−0.041	−0.334	0.266	−0.323	−0.287	−0.216
*p*-value	0.894	0.265	0.379	0.281	0.341	0.480
Chronic phase						
Pearson *r*	−0.107	−0.177	−0.002	−0.442	−0.142	−0.534
*p*-value	0.728	0.564	0.995	0.131	0.643	0.060

*Acute phase: 6-OHDA + vehicle, *n* = 12; 6-OHDA + LD, *n* = 13. Chronic phase: 6-OHDA + vehicle, *n* = 12; 6-OHDA + LD, *n* = 13. Highlighted values denote significant correlation*.

**Table 2:** Correlation analysis between A9/A10 lesion extent and difference in time in levodopa (LD) box between pre and post-conditioning sessions in the conditioned place preference (CPP).

	**Lesion extension**					
**Difference in time LD box**	**A9 lesion**	**A9 lesion right**	**A9 lesion left**	**A10 lesion**	**A10 lesion right**	**A10 lesion left**
Acute phase						
Pearson *r*	0.199	−0.030	0.315	0.477	0.388	0.443
*p*-value	0.461	0.912	0.235	0.062	0.138	0.086
Chronic phase						
Pearson *r*	0.281	0.303	0.182	0.442	0.469	0.314
*p*-value	0.311	0.272	0.517	0.099	0.079	0.254

*Acute phase: 6-OHDA, *n* = 16. Chronic phase: 6-OHDA, *n* = 15*.

## Discussion

In the present work, we aimed at assessing the behavioral consequences of LD administration in an animal model of PD, with a focus on reward and impulsivity. Our model was characterized by a significant anterior-posterior bilateral dopaminergic degeneration in both A9 and A10 regions. Importantly, the extent of our lesions presented some degree of variability between experimental subjects. In addition to the dopaminergic degeneration in A9 and A10 regions, we also observed a significant reduction in DA content in their major projection targets, dSTR and NAcc, respectively. As expected, 6-OHDA injections caused significant motor impairments in skilled forelimb function in lesioned animals. In parallel, we found that LD had a significant impact on impulsive-like behavior. In the training phase of the VDS task we first observed that 6-OHDA lesioned animals presented a significantly smaller percentage of premature responses across sessions. This might reflect differences in learning (and not in impulsivity), as the curves converged at the end of the training period. Indeed, at the 3 s phase of the VDS test day no differences were observed. However, in the test day we observed, in both acute and chronic phases, a significant increase in the number of premature responses as the delay to response was increasing. Additionally, our results showed that therapeutical doses of LD further accentuated delay intolerance, as seen by the increase of premature responses of LD-treated animals, compared to their vehicle controls. In a previous report, premature responses in this task were found to be sensitive to the effects of impulsivity-inducing drugs (Leite-Almeida et al., 2013). In the acute phase, the effects of LD administration on increasing premature responses in the 12 s delay were seen in sham animals, with 6-OHDA animals showing a trend for increased premature responding. In turn, chronic LD treatments significantly increased the number of premature responses in 6-OHDA animals, also in response to larger delays. We ruled out the possible implication of increased motor activation caused by LD on our results by quantifying the latency to feed, which showed no differences between groups during either phase.

In the clinical context of human PD, the use of DAg has been more strongly linked to the development of ICD than LD (Driver-Dunckley et al., 2003; Weintraub et al., 2006). Interestingly, there are cases of development of ICD only after introduction of DAg in patients previously on LD monotherapy (Klos et al., 2005). In line with this, previous experimental data have shown that a widely used DAg, pramipexole, increases impulsive responding in a striatum-lesioned model of PD, as seen by a significant increase in probability discounting (Rokosik and Napier, 2012). Based on this evidence, the interpretation of the LD effects we observed is not straightforward. However, it has been shown that a combined use of LD and DAg can increase the risk for the development of ICD when compared to DAg monotherapy (Weintraub et al., 2010), and that LD can increase delay intolerance—a construct that we also analyzed with our task—in PD patients (Cools et al., 2003). Although the exact mechanisms are currently not understood, these observations together with our own results, suggest that LD may play a role in the etiology of ICD. More recently, a study reported that in an animal model of PD with nigrostriatal degeneration, LD did not increase motor or waiting impulsivity (Engeln et al., 2016). However, it is important to highlight that unlike in the aforementioned study, in which dopaminergic lesions were restricted to the mesostriatal pathway, we employed here an animal model of PD with both mesostriatal and mesocorticolimbic degeneration. This difference could suggest that in PD a functional disruption in both mesostriatal and mesocorticolimbic pathways might be critical for the contribution of LD to the development of impulsive behaviors.

A large body of evidence has established strong links between disruptions in dopaminergic function and various forms of impulsivity (Dalley and Roiser, 2012). However, impulsive behaviors are not a typical manifestation of the parkinsonian personality (Dagher and Robbins, 2009). This supports the idea that although a parkinsonian condition is not sufficient for the manifestation of ICD in PD, the interaction between DRT and dysfunctional dopaminergic pathways might be one of the factors behind the development of these behavioral disturbances. Additionally, other factors such as impulsive personality or a previous history of ICD or substance abuse before disease onset, have also been shown to contribute to an increased risk of ICD in PD (Ceravolo et al., 2009). However, the effects of LD on impulsivity are not limited to the PD condition. Indeed, we also observed in our study that LD increased premature responding during the VDS task in sham-lesioned animals. This result is in line with several studies showing that the effects of dopaminergic stimulation with pramipexole can significantly increase impulsive responding in non-lesioned rats (Madden et al., 2010; Johnson et al., 2011; Rokosik and Napier, 2012; Engeln et al., 2016), and that LD can increase impulsivity in healthy humans (Pine et al., 2010). Put together, the results of the studies discussed above and ours, suggest that LD may thus hold a generalized disruptive impact upon neural networks responsible for impulse control. In the particular case of PD, such effects might interact in specific ways with a widespread functional disruption in dopaminergic signaling and lead to ICD. However, the exact mechanisms by which different classes of DRT might affect each of the dopaminergic pathways that are differently affected in PD, and later contribute to ICD, remain to be elucidated.

Human evidence has also shown that a prolonged duration of DRT treatment in PD patients is associated, among other factors, with an increase in novelty-seeking scores, which can underlie the development of impulsive behaviors that characterize ICD (Bódi et al., 2009). However, our results from the VDS test, and the comparison we have established between each individual performance in the acute and chronic phase of the test did not show a significant increase in premature responding as a result of prolonged LD treatment.

In addition to the effects of LD on impulsivity, we investigated the rewarding effects of LD in 6-OHDA-lesioned animals. In our study, we observed that neither acute nor chronic LD treatments were able to condition 6-OHDA-lesioned animals in the CPP paradigm. Previous studies have shown that an acute LD treatment holds rewarding properties in SNc-lesioned rodents overexpressing α–synuclein (Engeln et al., 2013b); however, in another study LD failed to condition a rat model of PD with bilateral SNc/VTA model of PD (Zengin-Toktas et al., 2013). Once more, the way dopaminergic function was differently affected by the lesion strategies employed in each one of the studies—SNc specific in the former and medial forebrain bundle lesions in the later—could offer some explanation for the observed discrepancies. Indeed, recent evidence showed that the rewarding effects of several dopaminergic drugs were associated with DA degeneration in the posterior, but not anterior, VTA (Ouachikh et al., 2013, 2014), and that medial forebrain bundle dopaminergic lesions increase sensitivity to the rewarding effects of apomorphine, measured with CPP (Campbell et al., 2014). Human studies have also shown that in PD patients reward responsiveness was positively correlated with disease severity, measured with DAT binding (Aarts et al., 2012). These results seem to suggest that the rewarding effects of LD may depend on the dopaminergic degeneration profile caused by PD-mimicking lesions; however, more recent evidence reveals this may be insufficient to fully explain LD-induced reward. For instance, in relation to the study by Engeln et al. (2013b), in two studies employing a similar lesion approach and higher LD doses administered chronically, this drug failed to induce CPP (Loiodice et al., 2017a,b).

Currently, the underlying mechanisms for the rewarding effects of LD, are still poorly understood but they have been proposed to stem from the repetitive overstimulation of the brain reward system, which over time becomes hypersensitized (Voon et al., 2017). Functional imaging studies performed in PD patients with DDS/ICD support such hypothesis. On the one hand, it has been shown that these patients display an increased LD-induced DA release in the ventral striatum, which was correlated with subjective LD craving (Evans et al., 2006). On the other hand, additional studies have reported an increased ventral striatal activation and DA release in response to reward-related stimuli (Frosini et al., 2010; O’Sullivan et al., 2011). These ventral striatal changes in dopaminergic signaling may be related with the effects of LD on dopaminergic function. Indeed, it has been proposed that as PD progresses, LD may cause DA release in a more pulsatile fashion (Grace, 2008). This continuous, pulsatile DA stimulation, mediated by high doses of LD continuously administrated in PD patients, is reminiscent of the physiological mechanisms that are believed to be responsible for the psychostimulant effects of drugs of abuse (Wanat et al., 2009), and could in turn mediate the rewarding effects of LD administration (Voon et al., 2017). Moreover, it is becoming increasingly acknowledged that the specific contribution of the DA system in drug addiction varies according to different classes of drugs of abuse (Pierce and Kumaresan, 2006). In this sense, it is important to highlight that the specific effects of LD on the complex neuronal networks that organize the brain reward system, particularly in the parkinsonian context and as disease progresses, are still poorly understood. Moreover, and given that in human PD LD therapy is commonly given in combination with other dopaminergic drugs, the rewarding effects of LD might be caused or exacerbated by synergistic effects with other DRT. In line with this, two studies showed that chronic LD exposure accentuated the rewarding effects of pramipexole administration, supporting the synergistic action of both dopaminergic drugs in the development of medication-induced conditioned responses in animals with PD lesions (Loiodice et al., 2017a,b). Alternatively, or in combination with these synergistic effects, individual vulnerability could also contribute for the reinforcing effects of LD (O’Sullivan et al., 2009). In this study, we looked for anatomical correlates that could offer a possible explanation for higher individual conditioned responses to LD in PD subjects. Our CPP analysis did not show any association between increased time spend in LD box and lesion extent in either A9 and A10, thus suggesting the lack of a contribution of lesion extent for increased susceptibility to any reinforcing effects of LD. However, a previous report has already raised the point that individual predisposition for the development of reward-seeking behaviors could help explain exceedingly high levels of pramipexole self-administration in PD rats (Engeln et al., 2013a). The sample size of these high responders was too limited to draw strong conclusions so future studies should further address this hypothesis.

The results of the present study add evidence to a growing body of literature linking the use of DRT in PD and the development of the behavioral complications that characterize ICD/DDS. In particular, we present evidence that in a rat model of PD with bilateral A9 and A10 lesions, LD treatment can induce the emergence of impulsivity-like behaviors, particularly after extended exposure. Nowadays, we are becoming increasingly aware of the complex dynamic changes that the progressive nature of DA degeneration in PD imposes on reward and impulse control brain networks, as well as the way DRT act upon these networks. In the future, experimental studies characterizing, in models of PD, the functional dynamics of DA signaling in key brain regions implicated in reward and impulse control, and following the long-term impact of DRT on these same regions, will contribute to a better understanding of the pathophysiological roots of DDS/ICD in PD.

## Author Contributions

MMC: study design, acquisition, analysis and interpretation of data, draft and final revision of the article. FLC, MM, CS-C, NK and CD: acquisition of data and final revision of the article. HL-A: interpretation of data and final revision of the article. NS and AJS: draft and final revision of the article.

## Conflict of Interest Statement

NK has received speaker’s fees, consultancy honoraria and travel support from Janssen-Cilag, Lundbeck, Sanofi-Aventis, Medochemie Generics and Elpen S.A. CD has received speaker’s fees and travel support from Boehringer-Ingelheim and Janssen-Cilag. The other authors declare that the research was conducted in the absence of any commercial or financial relationships that could be construed as a potential conflict of interest.
